# Threading plasmonic nanoparticle strings with light

**DOI:** 10.1038/ncomms5568

**Published:** 2014-07-28

**Authors:** Lars O. Herrmann, Ventsislav K. Valev, Christos Tserkezis, Jonathan S. Barnard, Setu Kasera, Oren A. Scherman, Javier Aizpurua, Jeremy J. Baumberg

**Affiliations:** 1 Cavendish Laboratory, NanoPhotonics Centre, Department of Physics, University of Cambridge, JJ Thompson Avenue, Cambridge CB3 0HE, UK; 2 Donostia International Physics Center DIPC and Centro de Física de Materiales CSIC-UPV/EHU Paseo Manuel de Lardizabal 5, 20018 Donostia-San Sebastián, Spain; 3 Department of Materials Science and Metallurgy, Charles Babbage Road, University of Cambridge, Cambridge CB2 30FS, UK; 4 Melville Laboratory for Polymer Synthesis, Department of Chemistry, University of Cambridge, Lensfield Road, Cambridge CB2 1EW, UK

## Abstract

Nanomaterials find increasing application in communications, renewable energies, electronics and sensing. Because of its unsurpassed speed and highly tuneable interaction with matter, using light to guide the self-assembly of nanomaterials can open up novel technological frontiers. However, large-scale light-induced assembly remains challenging. Here we demonstrate an efficient route to nano-assembly through plasmon-induced laser threading of gold nanoparticle strings, producing conducting threads 12±2 nm wide. This precision is achieved because the nanoparticles are first chemically assembled into chains with rigidly controlled separations of 0.9 nm primed for re-sculpting. Laser-induced threading occurs on a large scale in water, tracked via a new optical resonance in the near-infrared corresponding to a hybrid chain/rod-like charge transfer plasmon. The nano-thread width depends on the chain mode resonances, the nanoparticle size, the chain length and the peak laser power, enabling nanometre-scale tuning of the optical and conducting properties of such nanomaterials.

## Abstract (web summary)

Optical assembly of nanoparticle structures could open new avenues for manufacturing nanomaterials and devices. Herrmann *et al.* show the plasmon-induced laser threading of gold nanoparticle strings, enabling them to fabricate precisely assembled 12-nm wide conducting chains.

## Graphical abstract

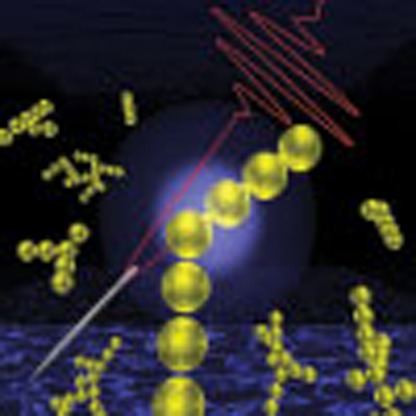

Although optical nanomaterials could find numerous potential applications^1^,^2^,^3^,^4^,^5^,^6^, controlling their fabrication at the nanoscale has proved challenging, particularly for scalable production, which precludes top-down fabrication. Although bottom-up approaches can produce simple nanoparticle (NP) geometries (such as particles or nanowires), more complex nanostructures have been hard to access reliably, emphasizing the need for a new technology. Current approaches have focussed on chemically binding NPs together or fusing individual nanocrystalline particles along common crystalline axes (oriented attachment)^7^,^8^, both of which have been difficult to control at the nanoscale and provide little control of contact conductivities.

Just as light is used to characterize optical materials, light can also be used to build them. Ultrafast lasers have so far been used to shape continuous metal surfaces with a variety of micro- and nanostructures such as needles, ripples, cavities, nanobumps and nanojets^9^,^10^,^11^,^12^,^13^,^14^,^15^. Nanojets in particular are columns of liquid material, frozen in the process of surging from the metal surface in the direction opposite to the incoming laser pulse. They are very similar to the back-jets observed on the surface of water following the impact of a pebble and are especially interesting as candidates for threading^16^. However, such nanojets have poor width control and cannot be aligned to face each other. These difficulties can be circumvented using surface plasmon resonances—the coherent oscillation of surface electrons. Plasmons enhance the optical fields and more importantly localize them to sub-wavelength regions termed hotspots. In dimers, or chains of plasmonic NPs, the plasmonic hotspots occur directly within the gaps^17^. Ultrafast lasers can therefore shape metals on the nanoscale to form a continuous thread in such dimers or chains of NPs.

Using ultrafast lasers to re-shape NP aggregates has been briefly explored^18^,^19^; however, only small numbers of welded NPs have been examined, while the crucial requirement is for a highly reproducible, high-yield fabrication process controlled in real time and monitored non-destructively. Such ultrafast re-shaping is very different from thermal laser-welding in nanowires^20^ or photochemically welded NP dimers^21^,^22^. Here we explore three essential questions. First, can we assemble nanomaterials by using light to selectively join NPs together on a large scale, and monitor the assembly in real time? Second, what are the important factors for this nanoassembly? And third, can we precisely control the dimensions of the connecting threads produced at the nanoscale?

## Results

### Assembling nanomaterials by using light

**Figure 1:**
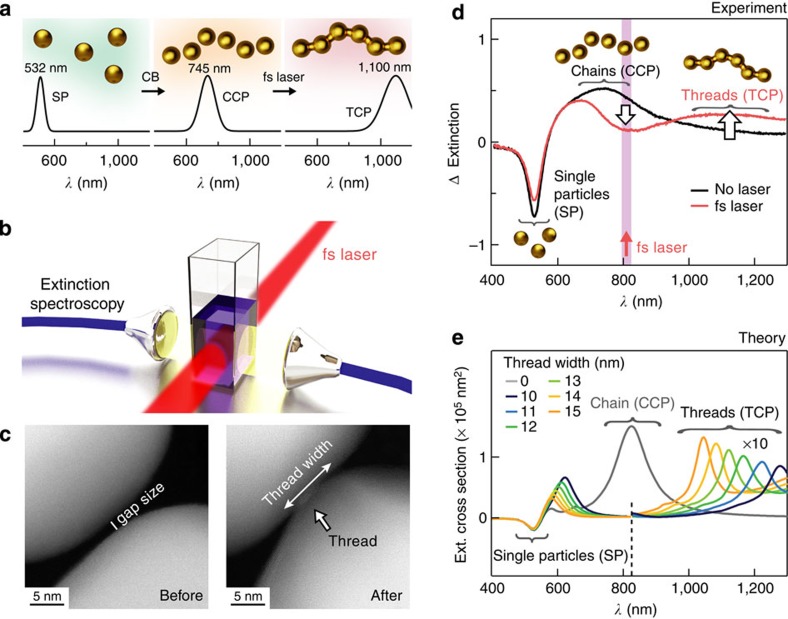
Laser processing of NP chains in water yields new plasmon mode. (**a**) Schematic process. Charge-stabilized gold NPs exhibit single particle plasmons (SP) at 532 nm resonance. Particles glued into chains by CB molecules give CCPs (745 nm resonance). Illumination with femtosecond laser pulses (schematic **b**) connects chains by metal thread into strings, producing TCPs (1,100 nm resonance). (**c**) TEM images of NP chain gaps before and after femtosecond irradiation. (**d**) After adding CB molecules to the NPs, spectra are different with/without femtosecond laser irradiation. Single NP response is subtracted from spectra. As the threads develop, a spectral dip around the laser wavelength and a peak at the rising TCP emerge. (**e**) Numerical simulations of resonant six-NP-long chains display TCP mode and indicate the range of nanothread widths contributing to the signal in **d**.

Starting from individually separated NPs, we find intense femtosecond laser pulses can fabricate thin threads to connect the NPs into continuous materials, on a large scale. Key to our advance is the initial chemical scaffolding of NPs into chains of plasmonic hotspots using barrel-shaped cucurbit[7]uril (CB) molecules, which glue together gold NPs with rigid 0.9 nm gaps^23^,^24^. Previously, we illustrated the precision of this interparticle control by achieving very reliable surface-enhanced Raman scattering (SERS) signals from molecules hosted within the CBs^23^. Aggregation of gold NPs with CB molecules produces large aggregates, whose structure is *a priori* unknown. Nevertheless, we showed that the optical behaviour of such large aggregates can be understood by decomposing the optical response into smaller one-dimensional chains embedded inside the aggregates^24^. These optical chain models help to understand the three spectral signatures, which allow us to monitor the threading in real time (summarized in Fig. 1a). First, the single plasmon resonance of 50 nm diameter gold NPs is situated at 532 nm. Second, coupling between the gold NPs mediated by CBs produces capacitive chain plasmon (CCP) resonances, at around 745 nm. Third, emerging at around 1,100 nm is the new threaded chain plasmon (TCP), which corresponds to an entirely conductive chain, with charge transfer allowed between neighbouring NPs. Processing by light induces large-scale threading, probed here across ml volumes (as depicted in Fig. 1b). Threads formed using unfocussed 805 nm, 200 fs, ultrafast laser pulses of 90 MW cm^−2^ intensity are directly seen in transmission electron microscopy (TEM) images (Fig. 1c and Supplementary Figs 1–3).

To clearly identify the emergence of new spectral features in the suspension of gold NPs, we subtract the background single plasmon response from the spectra to give the extinction change, ΔExtinction (Fig. 1d). As single NPs are captured in the chains being formed, a spectral dip is observed at 532 nm. The emerging CCP resonance originates from chains embedded in larger aggregates that are directly accessed by light polarized roughly parallel to them. Although such chains are kinked, this hardly affects the CCP, which depends instead on the number of NPs in each chain and their size^24^. Initially when CBs are added to the NP suspension in water, aggregation forms the CCP over ~5 min, completely reproducibly (Supplementary Fig. 4). This reproducibility is due to the precise subnanometre gaps, which control the field enhancements. As the latter are uniform along the chain, threads are formed on near-resonant femtosecond laser illumination, accompanied by the appearance of a new TCP resonance at around 1,100 nm. The spectral dip seen at the 805 nm laser wavelength is due to the transformation of aggregated chains into bridged conducting strings.

The emergence of the TCP in the near-infrared is clearly confirmed by numerical simulations (Fig. 1e). Here, perfectly spherical NPs of 50 nm diameter are assumed because, even though our NPs’ surfaces are slightly rough^25^, the measured optical response averages over a very large number of them. Plotting the extinction spectra for such NPs in strings of six (selected as their optical chain mode is then resonant at 805 nm, see below) produces near-infrared modes strongly dependent on the thread width. The observed 1,100 nm TCP peak (Fig. 1d) is thus attributed to a distribution of threads with widths around 12 nm. TEM (Fig. 1c) indeed confirms that bridging threads are only seen after irradiation and can be as small as 5 nm across. However, in such three-dimensional nanostructures, electron microscopy is unable to function optimally (Supplementary Figs 1–3). By comparison, spectroscopy is simple and unobtrusive, and following the near-infrared charge transfer TCP peak reveals the thread width across large-scale assemblies and in real time. Next, we examine the important factors for this laser-induced assembly.

### Important factors for assembling nanomaterials with light

In our experiments, threading is achieved with high peak power (*P*_peak_) ultrafast laser pulses, which indicates that the process is non-thermal. In a non-thermal process, the strong local electric fields can move around atoms (through optical forces) or ionize the lattice (weakening the bonds and increasing atomic mobility). To distinguish the effects of *P*_peak_ and average laser power (*P*_ave_), we varied the repetition rate while keeping *P*_ave_ constant at 500 mW (Supplementary Fig. 5). For both CW and 80 MHz illumination, neither the spectral hole on the CCP nor any TCP peak is observed; the effect on the optical chain mode spectrum is negligible. These data prove that it is not thermal heating (∝*P*_ave_) but field enhancements (∝*P*_peak_) that drive the bridging process. In addition, although thermal mechanisms should produce amorphous or single crystalline gold (depending on cooling rate), all our TEM data (Supplementary Figs 2 and 3) show polycrystalline NPs, indistinguishable from the unthreaded ones, with only little reconstruction to accommodate the thread. This evidence also strongly supports the athermal mechanism. The role of field enhancements is confirmed by the behaviour of the plasmon resonance.

The crucial importance of the plasmon resonance is seen by changing the NP size from 30 to 60 nm (Supplementary Fig. 6). Increasing their diameter tunes the CCP towards the laser wavelength. For 30 nm NPs, the chain plasmons have little spectral overlap with the laser and neither spectral hole nor TCP is seen. With increasing NP size, as the chain-mode field enhancements at 800 nm increase, both the spectral hole and the TCP peak become more pronounced, as discussed below.

**Figure 2:**
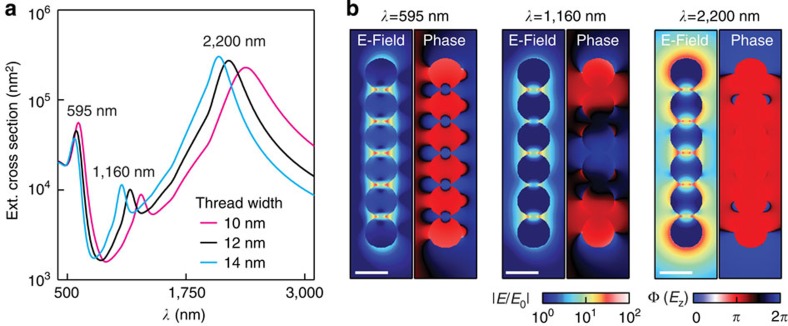
Near-infrared hybrid chain/rod-like mode. (**a**) Simulations identify three main peaks in the visible, near-infrared and mid-infrared. (**b**) Resonant field distribution (amplitude and phase) of the chain mode (595 nm), hybrid chain/rod-like mode (1,160 nm) and rod-like mode (2,200 nm). Scale bars, 50 nm.

The near-field distributions at the TCP show the laser-induced mixing of optical chain and rod-like modes (Fig. 2). The full extinction spectrum for thread widths from 10 to 14 nm (Fig. 2a) reveals three modes. The most intense peak in the mid-infrared (which is not possible to probe directly due to water absorption) is associated with a rod-like plasmon mode. This is not surprising because threading renders the entire chain conductive and transforms it into a string akin to a metal rod. The peak in the visible is associated with a modified optical chain plasmon mode, which remains because the bridges are very thin in comparison with the size of the NPs with field enhancements that are strong in the crevices around the thread.

The third peak is in the near-infrared, and the field and phase distributions (Fig. 2b) show that it is associated with a hybrid chain/rod-like mode. This hybrid nature is clearly seen in the field distributions for increasing thread thickness from 0 to 50 nm (that is, from chain to rod, Supplementary Fig. 7). In this mode, the electric field profiles exhibit both a rod-like envelope and local field enhancements in the interstices around the thread. This near-infrared hybrid chain/rod-like mode is situated in a very convenient spectral region that allows us to demonstrate excellent control of the nanothread width.

### Precise control over the dimensions of the threads

**Figure 3:**
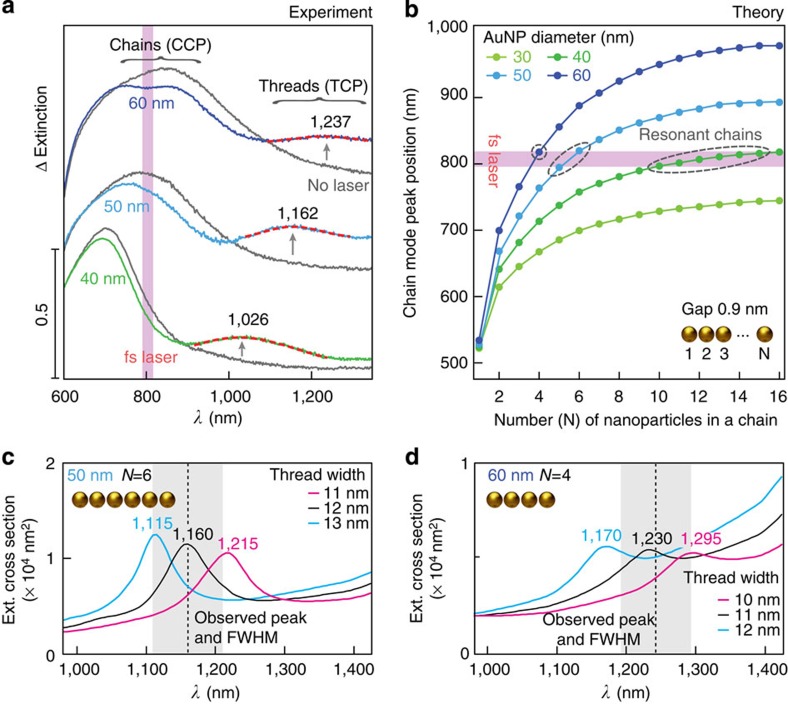
Control over the thread thickness to <2 nm. (**a**) Experimental differential extinction spectra recorded 40 min after introducing CBs into NP suspension. Chain plasmon tunes towards laser wavelength as NP size increases. Results are shown for the lowest laser powers that yield a pronounced TCP peak. (**b**) Chain mode peak *λ*_p_ for increasing lengths at each NP size, highlighting overlap with the 805 nm laser. (**c**,**d**) Simulated spectra for 50 and 60 nm NPs formed into threads, for resonant chain lengths of six and four NPs, respectively. The grey shading depicts the FWHM of the experimentally observed resonance. The simulations show that thickness of threads in **a** are 12±2 and 11±2 nm for the 50 and 60 nm NPs, respectively.

As the TCP depends on the width of the threads (Fig. 1e) and threading depends both on *P*_peak_ and on matching the CCP with the laser wavelength, we can use these to control the thickness of the thread. At lower powers, the only locations above the critical threading threshold are at the chain hotspots. Adjusting the CCP position is done by varying the NP diameter. From the emerging TCP peak, we can fit to extract its wavelength, *λ*_p_ and full width at half maximum (FWHM) linewidth, Δ*λ* (Supplementary Fig. 8). For NP diameters of 40, 50 and 60 nm (Fig. 3a), we find narrowest resonances at lower laser powers. To understand the precision possible for control of the thread width, the length distribution of laser-excited chains must be considered. On aggregation with CB molecules, the gold NPs form aggregates consisting of NP chains of varying length, extending in all available directions^23^,^24^,^26^. For each NP diameter, different chain lengths give different chain resonances, but as the chains become longer, the redshift of plasmon peak position saturates (Fig. 3b). For 30 nm NPs, chains are never resonant at 805 nm, which explains why laser irradiation has no effect (Supplementary Fig. 6). Upon resonant irradiation, the entire chains are simultaneously threaded. This is evidenced by the fact that the chain mode wavelength shifts dramatically as soon as the first conductive contact is made, losing resonance with the pump laser. Thus, for 40 nm NPs, many chain lengths are resonant and so the TCP peak is also broad with Δ*λ*/*λ*_p_=20%. For 50 and 60 nm NPs, there are few resonant lengths hence the TCP peaks are narrower, Δ*λ*/*λ*_p_=9.3% and 8.6%, respectively.

Plotting simulated spectra for several thread widths (Fig. 3c), we find the observed spectral positions and FWHM for the 50 and 60 nm NPs correspond to nanothread widths of 12±2 and 11±2 nm, respectively. This level of precise processing control is unprecedented for such a large-scale experiment. In principle though, the optical control can reach even subnanometre dimensions, as we now demonstrate in the case of single dimers.

**Figure 4:**
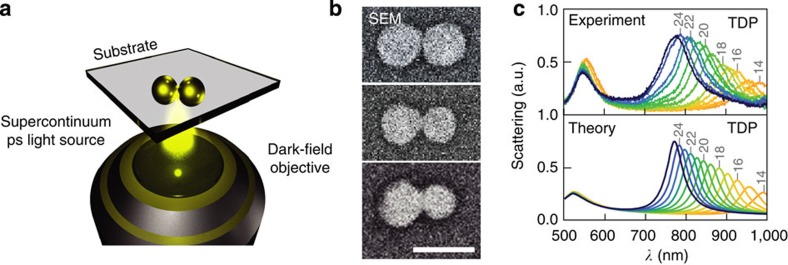
Pulsed irradiation to control thread width to ±1 nm in single dimers. (**a**) Single gold NP dimer illuminated for 0.5 s with a high-intensity bandpass-filtered ps supercontinuum laser. The broadband scattering spectrum is subsequently recorded with a low-intensity supercontinuum beam in a reflective dark-field geometry. (**b**) High-angle back-scattered scanning electron micrographs of a gold NP dimer with (top to bottom) a gap of ~1 nm and bridges of ~15 and ~30 nm (scale bar, 100 nm). (**c**) Gradual tuning of threaded dimer plasmon (TDP) in time after thread has been connected and is thickening, with irradiation tuned to be continually in resonance with this mode. Simulations (below) match red-shift of longitudinal mode as nanobridge thickens, while transverse mode is little affected. Thread widths are marked in grey.

Directing our attention on the shortest possible chain, we use a focused picosecond supercontinuum laser to controllably thread single gold NP dimers (Fig. 4). The optical scattering spectra (Supplementary Fig. 9) clearly show the submillisecond transition from the capacitive dimer plasmon to the threaded dimer plasmon (TDP). A dimer being the shortest possible chain, the latter is identical in this limit to the charge-transfer plasmon^27^. Once the threaded dimer plasmon forms (Fig. 4b), the thread width can be permanently adjusted through pulsed illumination with a bandpass-filtered source. In the optically accessible region below 1,000 nm, this enables us to fine-tune the bridge width with a relative error <0.2 nm (Fig. 4c), thereby showing that it is possible to create widely tuneable plasmonic materials with a well-defined optical response.

## Discussion

Although a variety of mechanisms are involved in optical construction of threads between NPs, the fact that our results depend on *P*_peak_ and not on *P*_ave_ allows us to identify two significant contributions. Non-thermal melting (dependent on *P*_peak_) occurs when the light–matter interaction lasts only for the pulse duration, before electrons can transfer any heat to the lattice. As a result, the excited electrons instantaneously weaken the lattice, causing local material melting^28^. Observations of such Si crystal melting report 150 fs timescales^29^. As our results depend on the surface plasmon resonance, we believe the lattice transiently weakens only at the surfaces situated directly across the gap between NPs. Also dependent on *P*_peak_ are the field enhancements in the gap between the NPs, which mediate plasmonically enhanced optical forces acting on mobile gold surface atoms at the hotspot edges, drawing them into the gap^30^,^31^. A second force on the atoms is produced by plasmonically enhanced emission of hot electrons, which induces local atomic charging that drives reconstruction of the nanogaps. Melting has indeed been reported in metal NPs for intensities of 10^10^ W cm^−2^ with theory suggesting it to be non-thermal^18^. Here, threads are obtained with thousand-fold smaller laser intensities of 2 × 10^7^ W cm^−2^, because we excite directly at the plasmonic hotspots, which we first carefully construct to profit from the near-field enhancement and thus strong local forces for materials processing.

To summarize, starting from NP chains in solution with precise gaps, we create large numbers of near-identical, continuous strings joining NPs with a conducting metal thread. The thickness of the thread depends on the laser wavelength, the peak power, the chain mode plasmon, the NP size and the chain length. Our results prove that very precise control of nanomaterials growth using light can be achieved at a large scale. Because of the dramatically enhanced fields in the crevices around the thread, excellent prospects exist for device application in photovoltaics, sensing and surface-enhanced Raman scattering. By further tailoring the light fields, entirely new structures can be created. For instance, using light with azimuthal angular momentum, split rings and chiral strings can be realized, highlighting the strong potential for solution-processing-based metamaterials.

## Methods

### Sample preparation

Gold NP suspensions with nominal diameters of 30, 40, 50 and 60 nm (verified by SEM) at respective molar particle concentrations of 330, 150, 75 and 40 pM were used as received (British Biocell International). Their specified relative s.d. in size is below 8%. CB was synthesized according to the method published by Day *et al.*^32^ Suspended gold NP aggregates: all glassware were cleaned with aqua regia. A 1-mM CB stock solution was prepared (5.8 mg with 5 ml of Millipore 18 MΩ H_2_O) and aggregation of NPs induced through injection of appropriate calculated amounts (0.25–5 μl for 0.5–10 μM final CB concentration) of this solution into 500 μl of undiluted gold NP suspension inside a quartz cuvette. Gold NP dimers on coverslip: a mixture of 500 nM CB and 40 pM gold NPs was stirred for 20 s, then drop cast onto an indium-tin-oxide coated coverslip (SPI Supplies, 06463-AB) and dried at 60 °C. Gold NP dimers were identified using a Zeiss 1540XB Crossbeam SEM/FIB workstation and tagged with a nearby focused-ion-beam mark to aid subsequent location under the optical microscope.

### Extinction spectroscopy

The ultrafast pulses were generated from a Mira-pumped RegA system (150 fs at 800 nm, Coherent). The collimated laser beam was directed through an optical cuvette (Hellma QS 104) and onto a power meter (Coherent, Lasermate-Q). Light from a halogen lamp (OceanOptics DH-2000) was fibre-coupled (OceanOptics QP200-2-VIS-BX) through the cuvette perpendicularly to the laser beam and the transmitted light collected by a bifurcated fibre (OceanOptics QBIF50-VIS-NIR) and sent to two spectrometers (Ocean Optics QE65000, spectral range: 300–1,100 nm and NIRQuest512, 900–1,700 nm). The illuminated volume was 0.5 ml. A magnetic stirrer (Fisher Scientific 11-520-16S) underneath the cuvette holder (Thorlabs CBH100) ensured fast mixing after injection of CB. A schematic of the setup is shown in Supplementary Fig. 10.

### Power density values

For a pulsed laser source with an average power *P*_av_ and a repetition rate *ν*, the pulse energy is *E*_pulse_=*P*_ave_/*ν*. The peak power is therefore given by *P*_peak_=*E*_pulse_/*t*_pulse_, where *t*_pulse_ is the pulse duration. The peak power density (radiant flux density) is thus *P*_peak_/*A*, with *A* the beam area. For the experiments reported here, the repetition rate *ν*=250 kHz, the pulse duration *t*_pulse_=200 fs and the beam area *A*~0.18 cm^2^. The average power *P*_ave_ was adjusted with a half-wave plate and a linear polarizer to values between 0.1 and 1 W.

### Single particle scattering spectroscopy

The output of a supercontinuum laser (Fianium SC450-6, 450–1,700 nm output) is split into two beams with a beam splitter (10% reflection, 90% transmission). The high-intensity beam is sent through a tunable bandpass filter (Fianium Superchrome, central wavelength position between 400 and 850 nm and bandwidth 5–50 nm). The low-intensity beam passes through a variable neutral density (optical density 0.1–2.0) filter for further attenuation and recombines with the filtered high-intensity beam through an additional beam splitter. The recombined beam is then spatially filtered and sent through a KG2 filter to remove mid-infrared radiation. A circular beam block removes the central part of the beam. The remaining beam passes through the outer part of a water immersion objective lens (Leica HPX Plan APO × 63, 1.2 W) and focuses onto the upper surface of the sample coverslip. The beam obstruction ratio is chosen such that the incident beam undergoes total internal reflection at the coverslip surface. The scattered light is collected through the inner part of the microscope objective and fiber coupled (OceanOptics QP50-2-VIS-BX) into a spectrometer (OceanOptics QE65000, spectral range: 300–1,100 nm) for spectral analysis. In addition to the supercontinuum source, a halogen lamp in Koehler illumination can be employed for wide-field illumination of the object plane. A schematic of the setup is shown in Supplementary Fig. 11.

### Electron microscopy

TEM images of gold NP aggregates on a holey carbon film (Agar Scientific AGS147) were taken in a FEI Titan3 scanning transmission electron microscope operated at 300 kV, in which a small probe (~0.1 nm in diameter) was rastered across the sample and the scattered electron beam collected by an annular detector (annular dark-field imaging). With a sufficiently high inner collection angle (>60 mrad) of the high-angle annular dark-field image, the scattered intensity is largely incoherent (there are no contrast reversals) and intensity may be readily interpreted: when the atomic columns of a crystal are aligned with the electron beam, the bright peaks correspond to the atomic columns. Low Z elements or thin regions appear darker and the vacuum is black (zero intensity). A Hitachi S-5500 in-lens field emission scanning electron microscope was used to acquire scanning electron microscopy images of gold NP aggregates deposited on a p-doped silicon wafer (Sigma-Aldrich 647764) and gold NP dimers on an ITO-coated coverslip (acceleration voltage 3 kV).

### Numerical simulations

Far-field extinction spectra, near-field distributions and phase maps were calculated by numerical simulations using the full electrodynamic boundary-element method^33^,^34^. The method is based on the solution of Maxwell’s equations for inhomogeneous media characterized by a local dielectric function in terms of surface-integral equations evaluated at the interfaces. The electromagnetic field is then calculated in terms of the induced boundary charges and currents, which are obtained through discretization of the surface integrals and solution of the resulting matrix equations. A sufficiently large number of discretization points were chosen to ensure that the results are fully converged. A dielectric constant of 1.77 was assumed for water.

## Author contributions

Experiments were planned and executed by L.O.H., V.K.V. and J.J.B., with support for the chemical nanoassembly from S.K. and O.A.S. Simulations were performed by L.O.H., C.T. and J.A., while electron microscopy was performed by J.S.B. The data were analysed by L.O.H., V.K.V. and J.J.B., and all authors contributed to the manuscript.

## Additional information

**How to cite this article:** Herrmann, L. O. *et al.* Threading plasmonic nanoparticle strings with light. *Nat. Commun.* 5:4568 doi: 10.1038/ncomms5568 (2014).

## Supplementary Material

Supplementary Information Supplementary Figures 1-11 and Supplementary References
