# Supplementary material for: Threading plasmonic nanoparticle strings with light
Source: Nat Commun. 2014 Jul 28;5:4568. doi: 10.1038/ncomms5568 (PMC4124895; doi:10.1038/ncomms5568)
Supplement: Supplementary Information — Supplementary Figures 1-11 and Supplementary References [file ncomms5568-s1.pdf]

Supplementary Figures

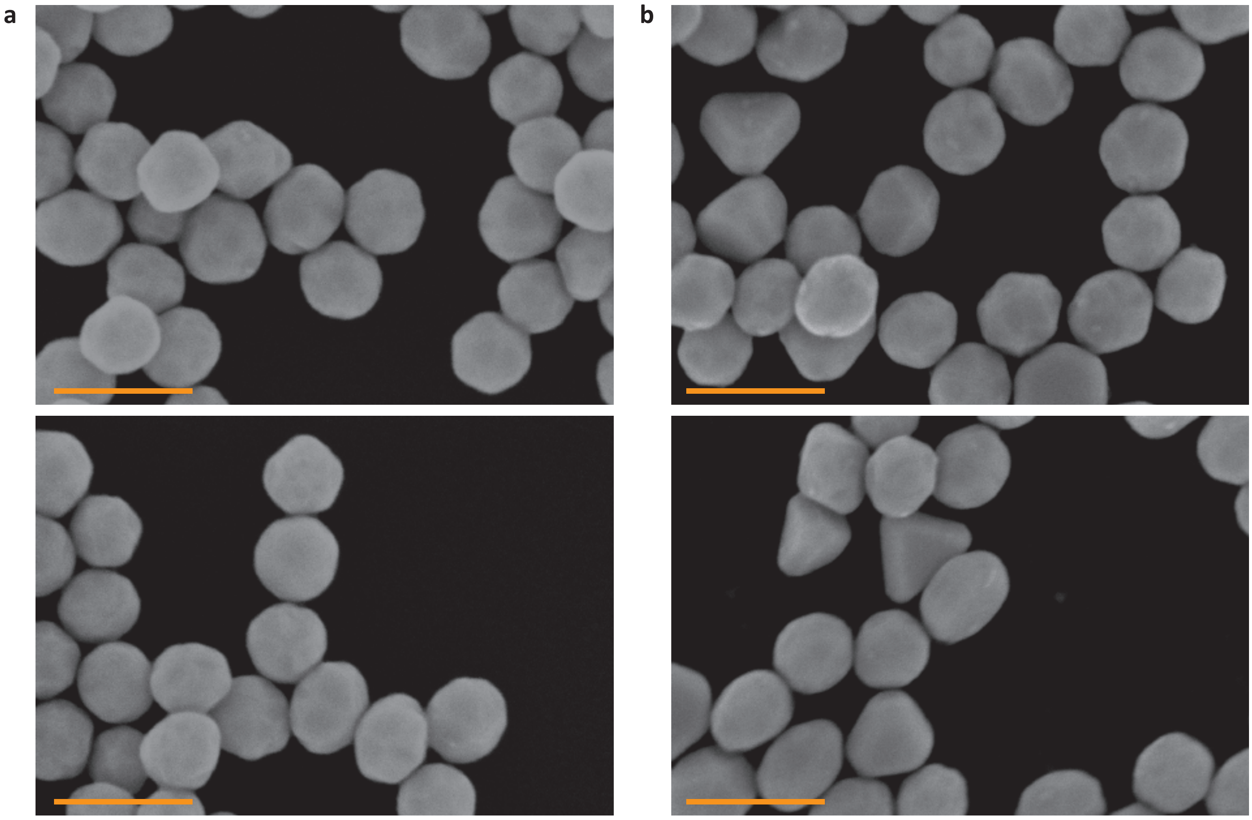

Supplementary Figure 1: Scanning electron microscopy (SEM) images of irradiated and non-irradiated gold nanoparticle (NP) clusters. High magnification SEM images of 40 pM 60 nm diameter gold NPs which were aggregated with 5 μM cucurbit[7]uril (CB[7]) over 45 minutes in **a**, the presence and **b**, the absence of a 200 fs pulsed 805 nm laser with a repetition frequency of 250 kHz (power density 56 MW cm^{-2}). No apparent differences between the samples can be observed at these magnifications, despite their enormously contrasting optical response, as the nanobridges are only 0.9 nm long (scale bars, 100 nm).

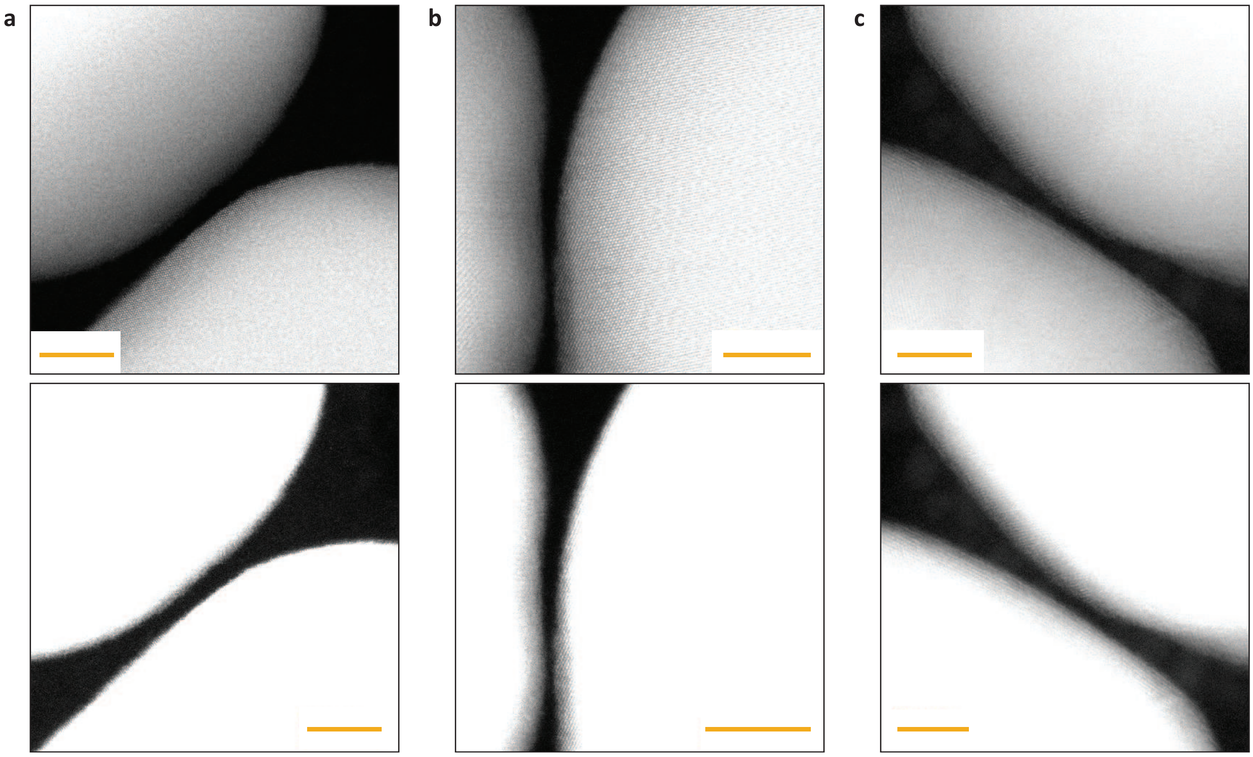

Supplementary Figure 2: Transmission electron microscopy images of non-irradiated gold nanoparticle (NP) clusters. A control sample containing 50 nm gold NPs was aggregated with 7.6 μM cucurbit[7]uril (CB[7]) for 45 mins in the absence of laser light. **a-c**, Representative high magnification images of the gap between two NPs show the confinement of the interparticle distance due to the presence of the molecular linker CB[7]. The images in the bottom row are a high contrast version of the images in the top row to enhance visibility of gap dimensions (scale bars, 5 nm).

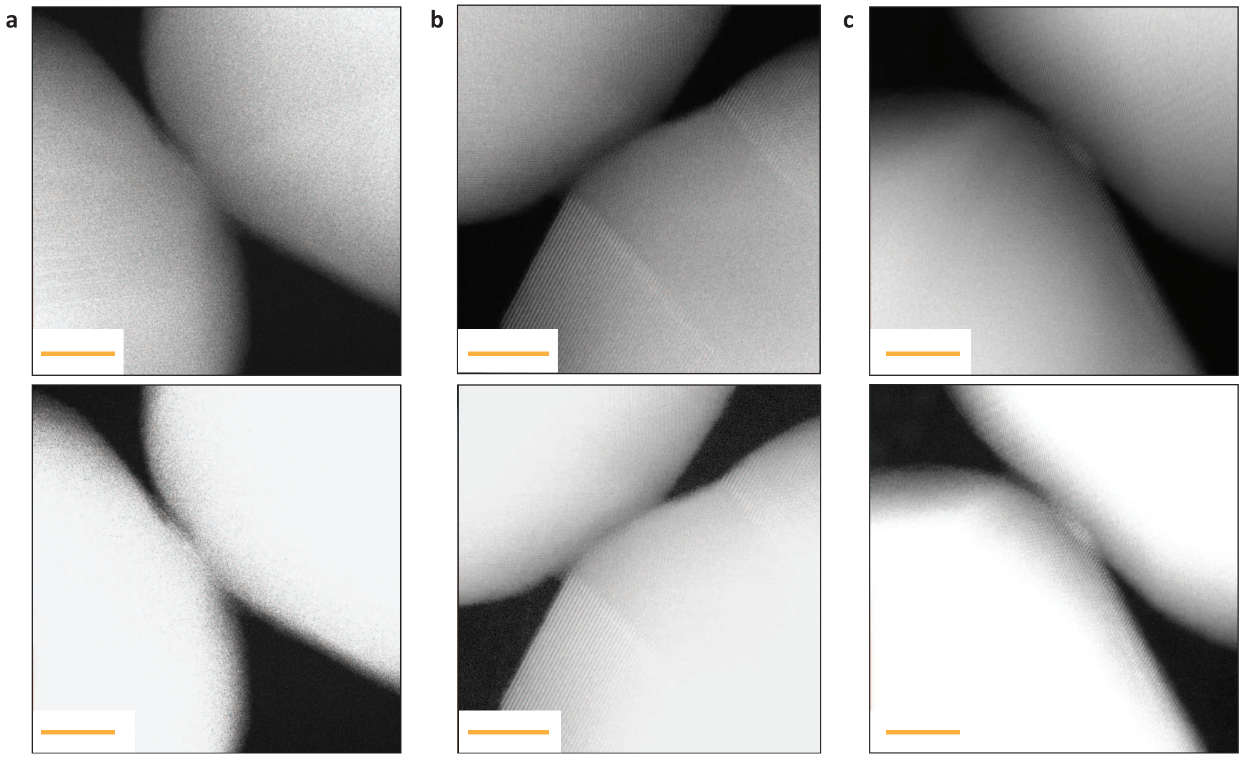

Supplementary Figure 3: Transmission electron microscopy (TEM) images of laser threaded gold nanoparticle (NP) clusters. A sample containing 50 nm gold NPs was aggregated with cucurbit[7]uril (CB[7]) for 45 mins while irradiated with 200 fs laser pulses at 805 nm with a repetition rate of 250 kHz and 500 mW average power (power density 56 MW cm^{-2}). **a-c**, NP chains which are resonant at 805 nm are connected by a thread with a diameter of order $\sim$ 10 nm. The images in the bottom row are a high contrast version of the images in the top row to enhance visibility of gold atoms in the gap. However, generally speaking, the imaging is rendered difficult because of the three-dimensionality of the gaps, where one particle can cast a shadow on the other. Additionally, the electron beams in SEM and TEM can affect the shape of the NPs thereby introducing artefacts. Moreover, the sample preparation, which requires depositing the aggregates on a substrate and drying them, inevitably diminishes the quality of the results as representative evidence for the actual optical experiment in solution (scale bars, 5 nm).

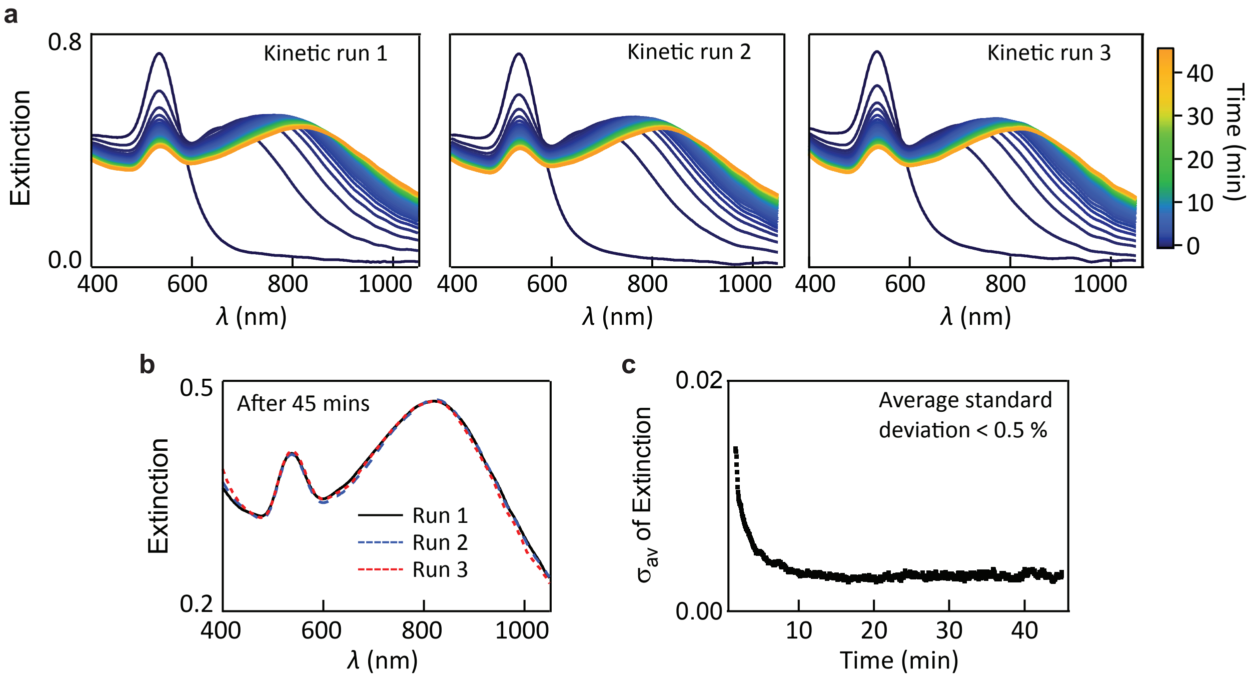

Supplementary Figure 4: Reproducibility of gold nanoparticle (NP) aggregation with cucurbit[7]uril (CB[7]). **a**, Aggregation kinetics of 60 nm gold NPs with 5 μM CB[7] over 45 minutes for three different runs. The time interval between each spectrum is 25 s. The extinction spectra clearly show how the initial single particle resonance reduces in intensity as the nanoparticles undergo aggregation, as well as the emergence of the capacitive chain plasmon (CCP) resonance.[1, 2] **b**, The extinction spectrum of each run after 45 mins. **c**, The average standard deviation (s.d.) of the extinction spectra between the three measurements as a function of time. It approaches a value of less than 0.5% after about 10 mins, verifying the excellent reproducibility of the aggregation process.

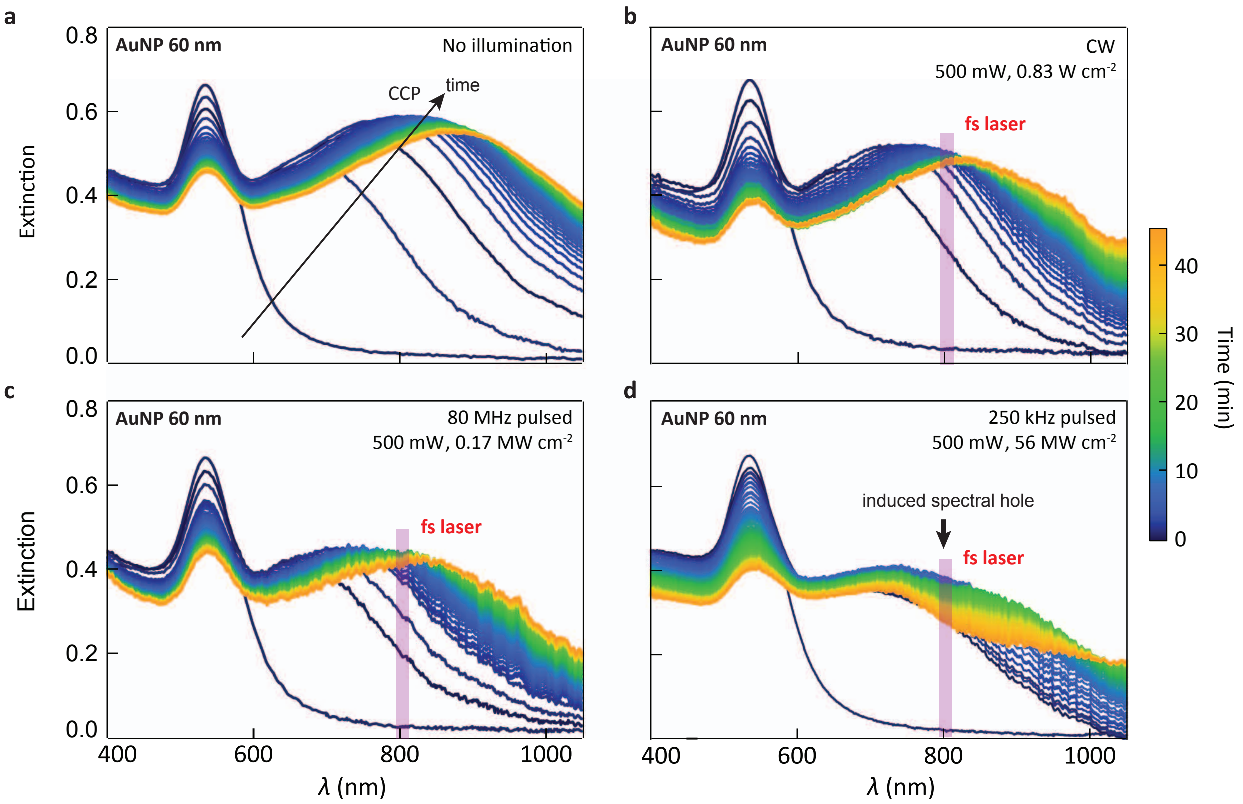

Supplementary Figure 5: Influence of laser repetition rate and pulse energy. Aggregation of 60 nm gold nanoparticles (NPs) with 5 μM cucurbit[7]uril (CB[7]) in **a**, the absence of laser illumination, and with a **b**, continuous wave (CW) laser (power density 0.83 W cm^{-2}), **c**, 200 fs pulses at 80 MHz repetition rate (peak power density 0.17 MW cm^{-2}) and **d**, 200 fs pulses at 250 kHz repetition rate (peak power density 56 MW cm^{-2}) at a wavelength of 805 nm. Laser is unfocused $\sim$ 0.18 cm^2 diameter beam, through cuvette. Only **d** shows laser-induced threading. This shows that it is the peak power P_{peak} which is relevant for the laser threading and not the average power P_{av}, indicating that it is a non-thermal process.

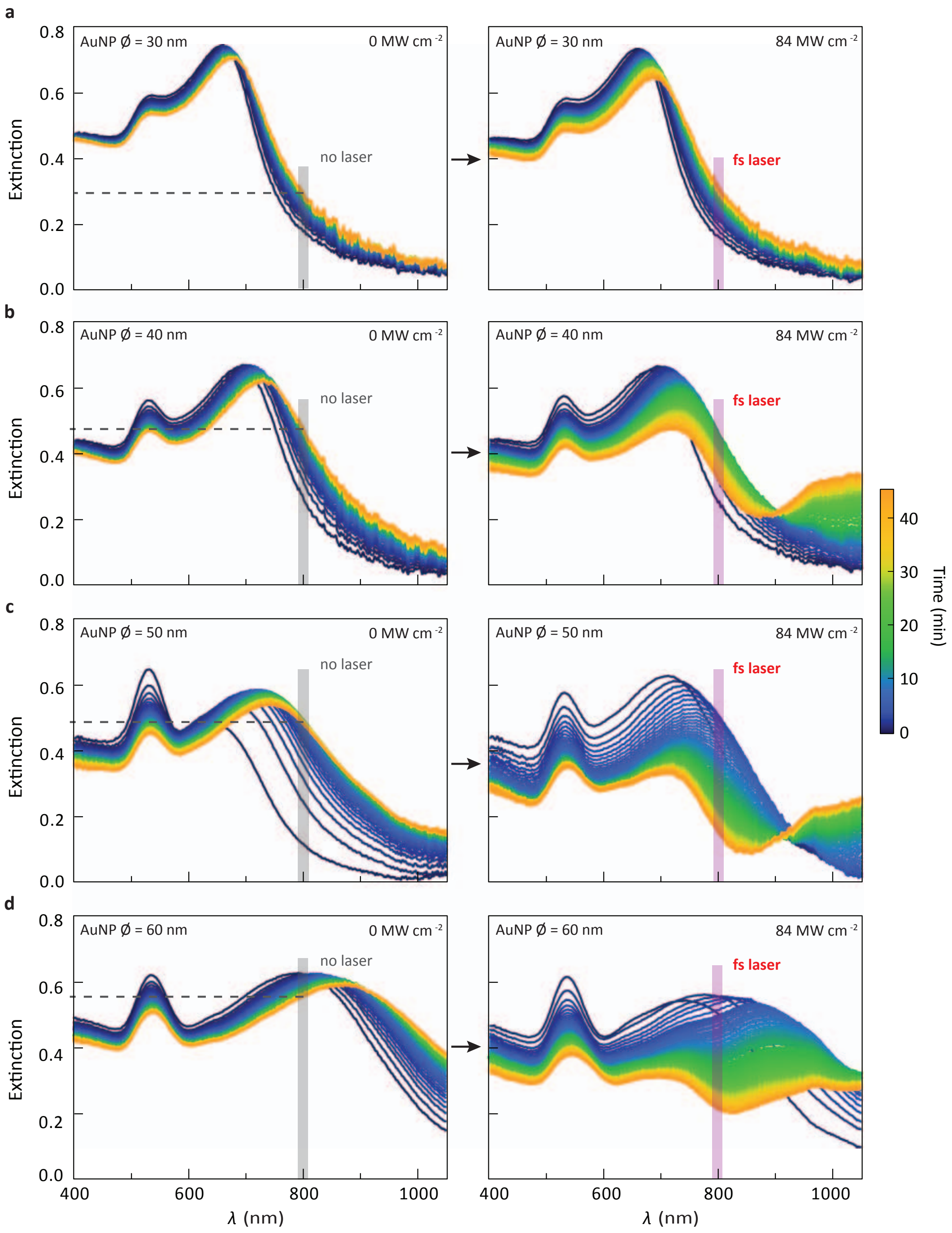

Supplementary Figure 6: Effect of nanoparticle (NP) diameter and position of capacitive chain plasmon (CCP) resonance on laser-threading. Aggregation kinetics of 30, 40, 50 and 60 nm diameter gold NPs (**a-d**) in the absence (left) and the presence (right) of fs pulsed laser illumination (power density 84 MW cm^{-2}, repetition rate 250 kHz, pulse length 200 fs, wavelength 805 nm). Laser-induced change in extinction increases with the overlap of the CCP mode with the laser at 805 nm. For all particle sizes, aggregation was carried out with a fixed ratio of 2.5 cucurbit[7]uril (CB[7]) molecules per nm^2 of gold NP surface.

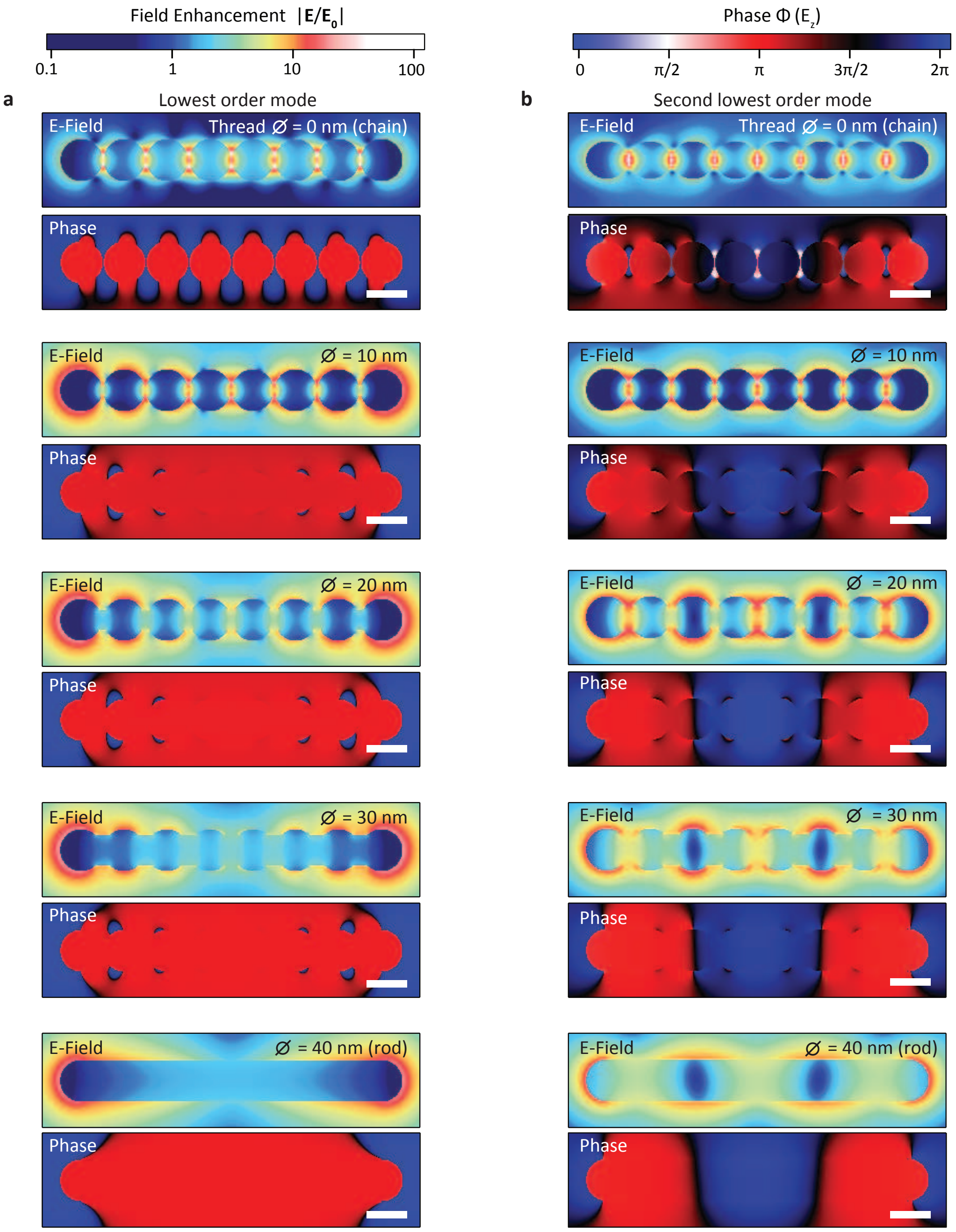

Supplementary Figure 7: Evolution of near-field and phase maps for stepwise increasing thread diameter from 0 nm (top, chain) to 40 nm (bottom, rod) in a chain of eight 40 nm diameter gold nanoparticles (NPs) separated by a distance of 0.9 nm. **a** shows the lowest order dipole mode and **b** the second lowest order mode which for threaded chains corresponds to the threaded chain plasmon (TCP) mode observed in the experiments (scale bars, 40 nm).

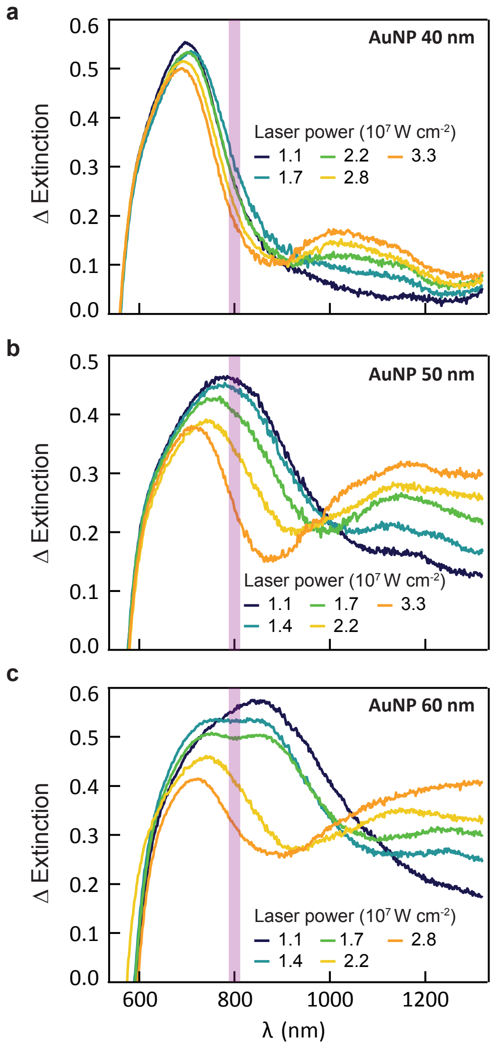

Supplementary Figure 8: Influence of power on thread populations. The effect of 200 fs pulsed laser illumination with a repetition rate of 250 kHz and power densities between 10-30 MW cm^{-2} on the extinction spectrum of gold nanoparticle (NP) aggregates after 45 mins. The gold NP diameter is **a**, 40 nm **b**, 50 nm and **c**, 60 nm. Power densities in the region $10 - 100$ MW cm^{-2} lead to the formation of a pronounced threaded chain plasmon (TCP) resonance peak whose position depends both on the gold NP diameter as well as the laser power.

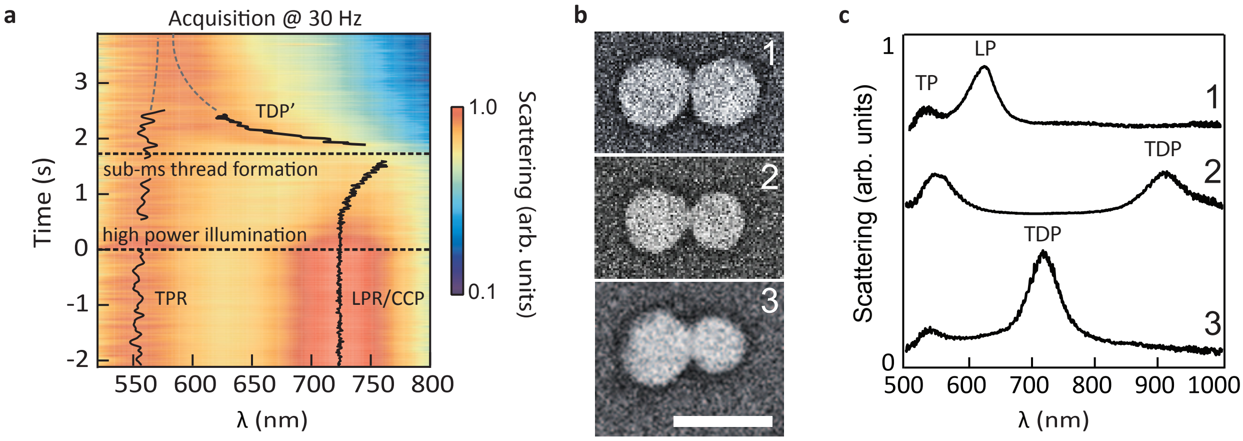

Supplementary Figure 9: Sub-millisecond threading of a two-particle chain. **a**, Scattering spectra of a gold nanoparticle (NP) dimer collected at 30 Hz with an integration time of 8 ms before ($t < 0$ s) and during high intensity white light illumination ($t > 0$ s). An initial red-shift of the longitudinal plasmon resonance (LPR) is followed by an abrupt jump and the emergence of a higher order charge transfer plasmon. **b**, High-angle backscattered electron microscopy images and **c**, the corresponding dark-field scattering spectra. The top (number 1) shows a gold NP dimer whose gap is confined to 0.9 nm by CB molecules. Clearly visible in the spectrum are the transverse plasmon resonance (TPR) at 545 $\pm$ 2 nm (uncertainty estimate from least squares fit of Lorentzian function) and the longitudinal plasmon resonance (LPR) at 628 $\pm$ 2 nm. Below (number 2 and 3) are two dimers connected by threads with diameters of 17 $\pm$ 2 nm and 29 $\pm$ 2 nm (estimated error from SEM image), respectively. The corresponding spectra now show the thread dimer plasmon (TDP) mode which sensitively depends on the width of the thread, but only weakly on its length. In the case of the thinner bridge, this mode occurs at 910 $\pm$ 2 nm, for the thicker bridge at 732 $\pm$ 2 nm. For those mode positions, theoretical simulations predict a thread diameter of 16.6 nm and 29.5 nm, in agreement with the values extracted from the SEM images.

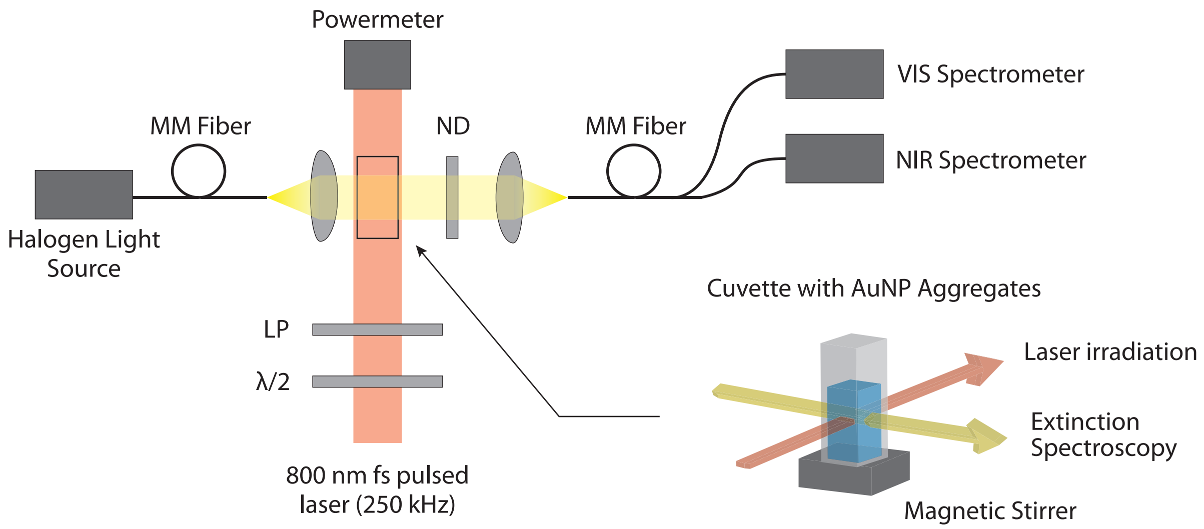

Supplementary Figure 10: Schematic of laser irradiation setup used to induce and monitor the thread formation between gold nanoparticles (NPs) in real time. A linearly polarized 805 nm pulsed laser (200 fs) with a repetition rate of 250 kHz is passed through a quartz cuvette such that it fully illuminates the contained sample volume. Its power is adjusted with a half-wave plate and a linear polariser. The induced change in the aggregation kinetics of the gold NPs is monitored based on the extinction of the light of a halogen lamp which is passed through the cuvette in an orthogonal direction.

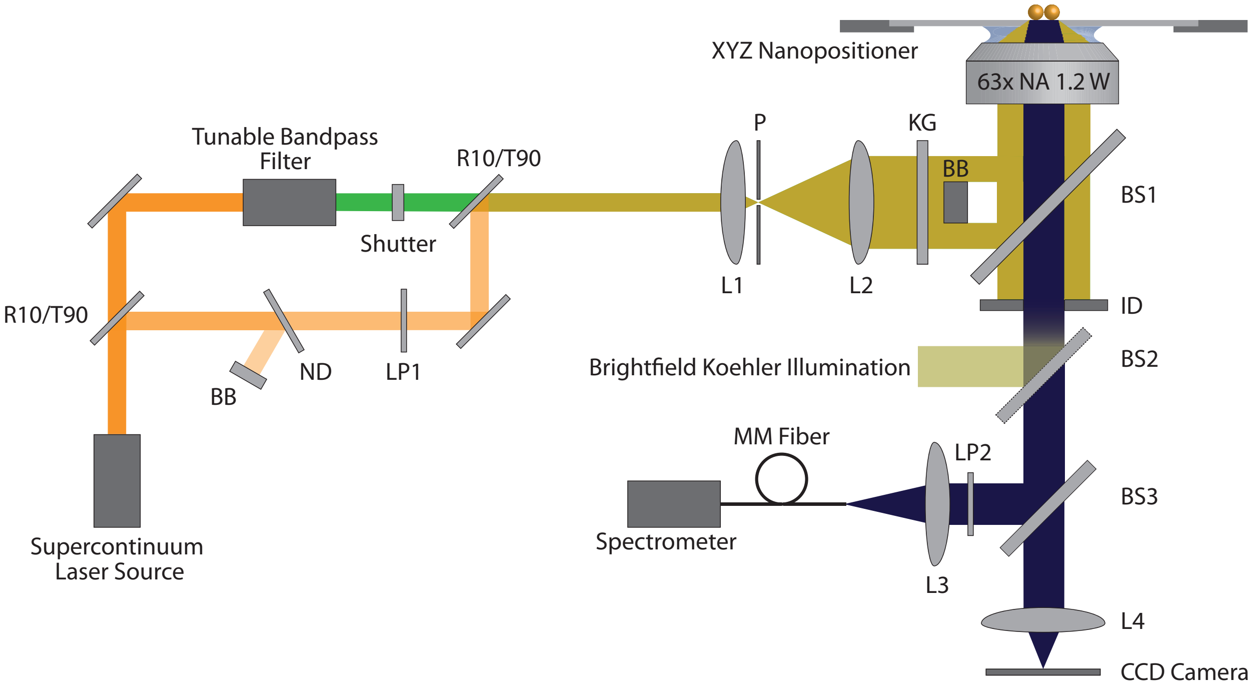

Supplementary Figure 11: Schematic of dark-field supercontinuum spectroscopy setup employed for the high-speed measurement and control of the thread formation in a NP dimer. The output of a supercontinuum laser (400-1700 nm) is split in two parts: Firstly, a high-intensity beam which passes through a tunable bandpass filter, used to induce thread formation. It can be controlled with a shutter placed after the bandpass filter. Secondly, a low-intensity beam to probe the scattering response. The intensity of this beam is further attenuated by a reflective neutral density filter. Both beams are recombined, spatially filtered and expanded, then focused through the outer part of a water immersion lens onto the nanoparticle dimer. The scattered light from the dimer is collected through the inner part of the objective (in a dark-field geometry) and sent to a spectrometer and a CCD camera for analysis. (Abbreviations: R10/90 denotes a beamsampler with 10% reflection, 90% transmission, BB = beam block, ND = neutral density filter, LP1 and LP2 are a linear polariser and analyser, L1-4 = achromatic lenses, KG = heat absorbing filter, BS1-3 = broadband beam splitters).

Supplementary References

[1] Taylor, R.W. *et al.* Precise subnanometer plasmonic junctions for SERS within gold nanoparticle assemblies using cucurbit[n]uril 'glue'. *ACS nano*, **5**, 3878–87 (2011).

[2] Esteban, R., Taylor, R.W., Baumberg, J.J., & Aizpurua, J. How chain plasmons govern the optical response in strongly interacting self-assembled metallic clusters of nanoparticles. *Langmuir*, **28**, 8881–90 (2012).
